# Sandwich type polyoxometalates encapsulated into the mesoporous material: synthesis, characterization and catalytic application in the selective oxidation of sulfides

**DOI:** 10.1039/c8ra03659d

**Published:** 2018-08-07

**Authors:** Elham Naseri, Roushan Khoshnavazi

**Affiliations:** Department of Chemistry, University of Kurdistan PO. Box 66135-416 Sanandaj Iran r.khoshnavazi@uok.ac.ir +98 87336224133 +98 87336224133

## Abstract

The A-type sandwich polyoxometalates of [(HOSn^IV^OH)_3_(PW_9_O_34_)_2_]^12−^ (P_2_W_18_Sn_3_) and [(OCe^IV^O)_3_(PW_9_O_34_)_2_]^12−^ (P_2_W_18_Ce_3_) were immobilized for the first time into the porous metal–organic framework MIL-101(Cr). FT-IR, powder X-ray diffraction, SEM-EDX, ICP analysis, N_2_ adsorption and thermogravimetric analysis collectively confirmed immobilization and good distribution of polyoxometalates into cages of MIL-101(Cr). The catalytic activities of the homogeneous P_2_W_18_Sn_3_ and P_2_W_18_Ce_3_ and the corresponding heterogeneous catalysts were examined in the oxidation of sulfides to sulfones with H_2_O_2_ as the oxidant at room temperature. The effects of different dosages of polyoxometalates, type of solvent, reaction time, amount of catalyst and oxidant in this catalytic system were investigated. The new P_2_W_18_Sn_3_@MIL-101 and P_2_W_18_Ce_3_@MIL-101 nanocomposites exhibited good recyclability and reusability in at least five consecutive reaction cycles without significant loss of activity or selectivity.

## Abstract 2

The A-type sandwich POMs of [(HOSn^IV^OH)_3_(PW_9_O_34_)_2_]^12–^ (P_2_W_18_Sn_3_) and [(OCe^IV^O)_3_(PW_9_O_34_)_2_]^12–^ (P_2_W_18_Ce_3_) were immobilized for the first time into the porous MIL-101 MOF. Their catalytic activities were examined in the oxidation of sulfides to sulfones at room temperature.

## Graphical abstract

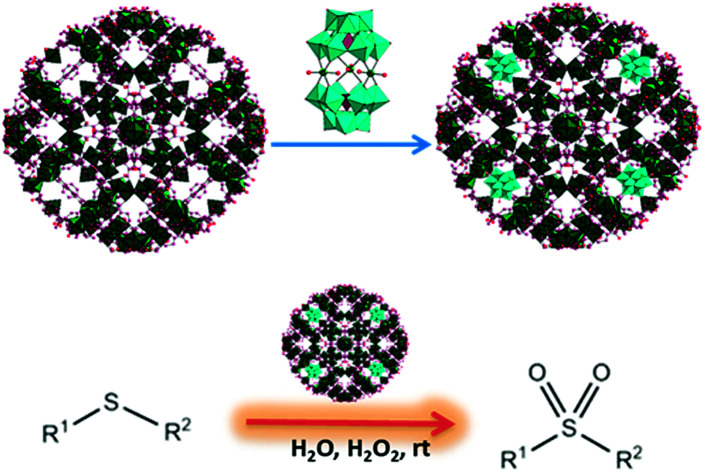

## 1. Introduction

Polyoxometalates (POMs) as a class of metal cluster complexes comprising transition metal oxide anions attract attention in light of their significant potential in medicine, structural chemistry, analytical chemistry, surface science, electrochemistry and photochemistry.^1–3^ Their utility for catalysis applications has been limited, notably due to their tendency toward low surface area (typically ≤ 10 m^2^ g^−1^) and porosity (lower than 0.1 cm^3^ g^−1^) of bulk POMs which hinder the accessibility of the active sites, together with a high solubility in polar solvents making it inconvenient for recovery and reutilize.^4–6^ In this sense, the heterogenization or immobilization of POMs onto various solid supports *via* dative,^7^ covalent,^8^ or electrostatic^9–11^ binding, such as silica, activated carbon, magnetic nanoparticles and titanium dioxide is highly desired to prepare new and robust heterogeneous catalysts, capable of being easily separated from a reaction mixture and recycled.^12–14^ Recently, metal–organic frameworks (MOFs) materials were also used for immobilization of POMs.^15^ Because of their structural features, MOFs relative to other porous matrices play an important role in the development of different catalysts, including those for enantioselective chiral reactions, asymmetric epoxidation of alkenes and allyl alcohols, oxidation of alcohols and in a synthesis of porous carbon materials through thermal decomposition of guest-free MOFs.^16–20^ MIL-101 family of materials (MIL: Materials of the Institute Lavoisier) are a very stable group of MOFs, result from the three-dimensional covalent connection of inorganic clusters and organic linkers. Its open-pore structure with the pores (∼3.5 nm) and pore windows (∼1.5 nm) are large enough to give access to voluminous reactant molecules diffusing into the pores. These properties and also able to be functionalized, accessible cages and very high specific surface area, make it an excellent candidate to support catalytic species.^21–25^

**Scheme 1:**
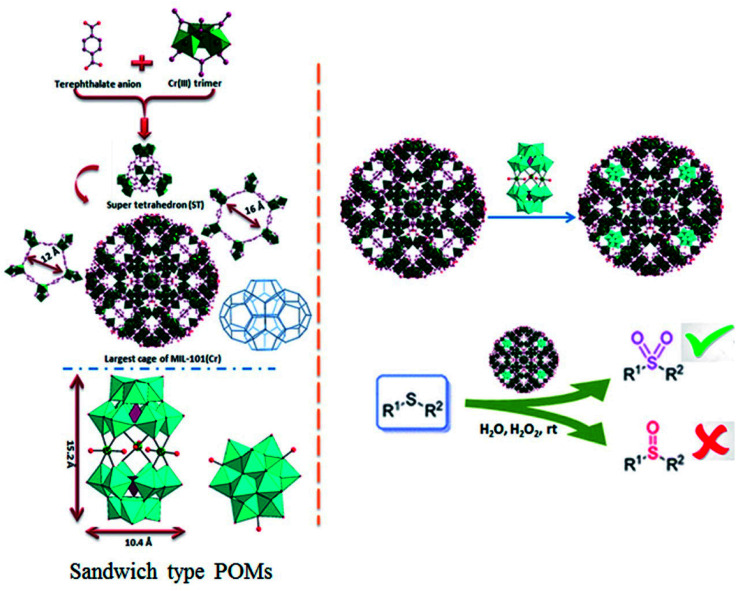
The synthesis pathway for preparation of nanocomposites.

In 2005, Férey *et al.* reported incorporation of the lacunary polytungstate of PW_11_O_39_^7−^ within the large cage of MIL-101(Cr).^26^ Following work by Férey *et al.,* numerous POM@MIL-101 composite materials containing various catalytically active polyoxometalates has been widely studied, and the resulting composites have been applied to H_2_O_2_-based alkene epoxidation, knoevenagel condensation, aldehyde–alcohol reactions, oxidative desulfurization, aerobic so on.^27–30^ Recently, a range of hybrid materials based on various Keggin polyoxometalates building blocks and copper organic frameworks with pyrazine derivatives have been discussed by Haddadi *et al.* and used as a heterogeneous catalyst for the selective oxidation of sulfides to corresponding sulfoxides and sulfones using H_2_O_2_.^31^ A-type sandwich polyoxometalate of K_11_H[(HOSn^IV^OH)_3_(PW_9_O_34_)_2_]·20H_2_O was immobilized for the first onto Nd-doped TiO_2_ nanoparticles as support material and these new nanocomposites used for the oxidation of different sulfides and alcohols.^32^ Herein we report the synthesis and application of new POM@MIL-101 composites materials containing A-type sandwich polyoxometalates in selective oxidation of sulfides. To the best of our knowledge, this is the first report about the immobilization of various potassium salts of [(HOSn^IV^OH)_3_(XW_9_O_34_)_2_]^12−^ (X = P and Si), [(OCe)_3_(XW_9_O_34_)_2_]^12−^ (X= P) and sodium salts [WM_3_(H_2_O)_2_(XW_9_O_34_)_2_]^12−^ (X = M = Zn and Co) into a MIL-101 metal–organic framework resulting in a series of POM@MOF composite materials in selective oxidation of sulfides. The structure of P_2_W_18_Sn_3_ and P_2_W_18_Ce_3_ consists of two A-type PW_9_O_34_ anions linked by three tin(iv) or cerium(iv) cations leading to A-type sandwich POMs (Scheme 1).

## 2. Experimental

### 2.1. Materials

All chemicals were used as received from different commercial sources. All the reagents used in the preparation of the composite materials, namely analytically pure chromium(iii) nitrate nonahydrate (Cr(NO_3_)_3_·9H_2_O, Aldrich), benzene-1,4-dicarboxylic acid (C_6_H_4_(CO_2_H)_2_, Aldrich), fluorhydric acid (HF, Merck) and DMF (*N*,*N*-dimethylformamide, Merck) were purchased and used without further purification. The chemicals used for desulfurization experiments, namely diphenyl sulfide, methyl phenyl sulfide, dimethyl sulfide, 2-(methylthio)ethanol, methyl 3-(methylthio)propionate, 2-furfuryl methyl sulfide, allyl sulfide, dibutyl sulfide (Fluka, 95%), acetonitrile (CH_3_CN, Merck) and hydrogen peroxide (H_2_O_2_, Merck 35%) were also used as received. The A-type sandwich polyoxometalates of K_11_H[(HOSn^IV^OH)_3_(PW_9_O_34_)_2_]·20H_2_O and K_9_(NH_4_)H_2_[(OCe^IV^O)_3_(PW_9_O_34_)_2_]·20H_2_O were prepared according to the previously described procedures.^33,34^

### 2.2. Characterization methods

The synthesized MOF and nanocomposites were characterized using; Infrared absorption spectra were recorded on a Bruker Vector 22 infrared spectrometer using KBr pellet method. Thermogravimetric analysis (TGA) was carried out under N_2_ flow while gradually increasing the temperature with a rate of 10 °C min^−1^, using a STA PT-1000 LINSEIS. The morphology of nanocomposites was revealed by a scanning electron microscope (MIRA3 FEG-SEM). The elements in the nanocomposite samples were probed with energy-dispersive X-ray (EDX) spectroscopy accessory to the MIRA3 FEG-SEM scanning electron microscopy. Nitrogen adsorption–desorption isotherms used to obtain the MIL-101(Cr) and POM@MIL 101(Cr) specific surface areas and pore volumes, calculated by the Brunauer–Emmett–Teller (BET) method. All as-prepared samples were degassed at 100 °C in vacuum for overnight prior to adsorption measurements. The experiments took place at −196 °C under variable relative pressure. The equipment was a Belsorp mini II adsorption analyzer (Bel-Japan) at −196 °C. Powder X-ray diffraction (XRD) analyses were collected at ambient temperature by a X'PertPro Panalytical, Holland diffractometer using a CuKα radiation (*λ* = 1.5418 Å). The accelerating voltage and applied currents were 40 kV and 30 mA, respectively. The C microanalyses was carried out with CHNS–O Elemental Analyzer Vario EL III, ELEMENTARY, Hanau-Germany while the amount of W and Cr were measured by inductively coupled plasma mass spectrometry (ICP-MS).

### 2.3. Preparation of the materials

#### 2.3.1. Solid support MIL-101(Cr)

The porous metal–organic framework (MOF) material MIL-101(Cr) was prepared hydrothermally, using a modified procedure of the method described by Férey *et al.*^26^ Benzene-1,4-dicarboxylic acid (H_2_BDC) (0.332 g, 2 mmol) was added in small portions to an aqueous solution of Cr(NO_3_)_3_·9H_2_O (0.8 g, 2 mmol in 10 mL of deionized water) and stirred at room temperature to obtain an homogeneous suspension for 20 min, then fluorhydric acid (0.1 mL) was added to the above suspension and stirred for 30 min. The dark blue-colored suspension was placed in a Teflon-lined autoclave (125 mL, Paar model 4748), for 9 h at a heating temperature of 220 °C without stirring. After slowly cooling to ambient temperature (inside the oven), the green powder was collected by repeated centrifugation and thorough washing with deionized water. Since the product was a mixture of MIL-101 powder and few amounts of needle-like crystals of recrystallized H_2_BDC. It was washed overnight with DMF under reflux against the removal of recrystallized H_2_BDC. Afterwards, to remove the DMF solvent, the resulting solids were washed with deionized water several times. The product was dried in an air oven at 70 °C overnight followed by soxhlet extraction in ethanol for 24 h. Samples were activated under vacuum 3 days at 70 °C with a high yield 83.8% based on chromium.

#### 2.3.2. P_2_W_18_Sn_3_@MIL-101 composite

The composite material P_2_W_18_Sn_3_@MIL-101 was prepared through the immobilization of the potassium salt of P_2_W_18_Sn_3_ in the porous solid support MIL-101 using a modified procedure of the method described by Balula *et al.*^35^ In the “impregnation” method, 0.0733 mmol of dry MIL-101 synthesized in an autoclave as described above was added to an aqueous solution of a moderate amount of P_2_W_18_Sn_3_ (0.25 mM, 0.5 mM, 1 mM, 2 mM, 4 mM; 15 mL) and the mixture was stirred at room temperature for 24 h. The solid separated by centrifugation and then washed several times thoroughly with deionized water and dried in an air oven at 60 °C overnight followed by activated under vacuum 3 days at 70 °C. Elemental analysis (w/w %): C, 33.5; Cr, 8.18; W, 21.52. Based on the elemental analysis results and molecular weight of K_11_H[(HOSn^IV^OH)_3_(PW_9_O_34_)_2_]·20H_2_O, W content, 57.98%; MW, 5708, we estimated the P_2_W_18_Sn_3_ content of the P_2_W_18_Sn_3_@MIL-101 materials to be approximately 65 μmol g^−1^ of dry powder, or 37 w/w %. The content of P_2_W_18_Sn_3_ was calculated according to the formula, μmol g^−1^ = 1 × 10^6^ (W content in the P_2_W_18_Sn_3_@MIL-101, %)/(MW of P_2_W_18_Sn_3_ × W content in the P_2_W_18_Sn_3_%).^28^

#### 2.3.3. P_2_W_18_Ce_3_@MIL-101 composite

The composite material P_2_W_18_Ce_3_@MIL-101 was prepared through the immobilization of the potassium salt of P_2_W_18_Ce_3_ in the porous solid support MIL-101 according to the preparation procedure of P_2_W_18_Sn_3_@MIL-101 composite unless, P_2_W_18_Sn_3_ was replaced by P_2_W_18_Ce_3_.

### 2.4. General test for the oxidation

A mixture of bulk polyoxometalates (P_2_W_18_Sn_3_ or P_2_W_18_Ce_3_) or composites (P_2_W_18_Sn_3_@MIL-101 or P_2_W_18_Ce_3_@MIL-101) (50 mg) as catalyst, 35% H_2_O_2_ aqueous solution (5.85 mmol) and solvent (2.5 mL) were placed in a 25 mL glass bottle. After 5 min, the substrate (1 mmol) was added under stirring. The reaction time was counted after the addition of sulfide, and then the reaction mixture was stirred at the experiment temperatures for the appropriate time. The sample was collected from the mixture at time intervals and then the progress of the reaction was followed by TLC (eluent: *n*-hexane/EtOAc, 3 : 1) and stopped when a complete conversion of the substrate was observed. The catalyst was filtered off at the end of reactions, washed several times with ethyl acetate followed by ethanol (4 × 5 mL), heated in an oven at 70 °C overnight and then reused using the same reaction conditions. The starting material and product are insoluble in water and it was used just as an environment for stirring. Therefore, the reaction mixture was transferred to a separating funnel and the product was extracted with CH_2_Cl_2_ (3 × 5 mL). After evaporation of organic layer, the crude products were recrystallized from hot ethanol and the pure products were obtained in 94–98% yield. Stability test of the P_2_W_18_Sn_3_@MIL-101 and P_2_W_18_Ce_3_@MIL-101 catalysts were carried out running six consecutive experiments.

## 3. Results and discussion

### 3.1. Synthesis

The MIL-101(Cr) is built up from a corner-sharing of so-called super tetrahedron (hereafter noted ST), which is formed by rigid chromium(iii) octahedral trimers (the vertices of the ST) and terephthalate anions (the edges of the ST) (see Scheme 1).

The connection between of the corners of the ST building blocks ensures a 3D cubic zeotype structure with two types of mesoporous cages (*∅* = 29 and 34 Å) (extended MTN topology), accessible through microporous windows (*∅* = 12 and 16 Å). The large cages possess both 12 Å pentagonal and 16 Å hexagonal windows with internal free diameters of 34 Å. Therefore, these cavities are sufficiently spaced to accommodate large guest molecules (such as the successful encapsulation of the sandwich-type anions, P_2_W_18_Sn_3_ or P_2_W_18_Ce_3_ (∼10.4 × 10.4 × 15.2 Å^3^)), into cages of MIL-101(Cr). The MIL-101(Cr) has been employed as a useful and versatile solid support for preparation of heterogeneous catalysts because of its open-pore structure with large and accessible cages.^26^ The incorporation of POMs in the mesoporous MIL-101(Cr) has been carried out by the anionic exchange between the counter-ions of the MIL-101(Cr) (nitrate ions coming from the Cr(NO_3_)_3_ precursor) and the negatively charged P_2_W_18_Sn_3_ or P_2_W_18_Ce_3_.^36^ For first time, MIL-101(Cr) with Cr_3_F(H_2_O)_2_O[(O_2_C)–C_6_H_4_–(CO_2_)]_3_·*n*H_2_O formula was synthesized by the HF–Cr(NO_3_)_3_–H_2_BDC–H_2_O system.^26^ The composites of the sandwich-type POM of P_2_W_18_Sn_3_ and P_2_W_18_Ce_3_ with MIL-101 were prepared by impregnation of the synthesized MIL-101 in an autoclave as described above in aqueous solution of P_2_W_18_Sn_3_ or P_2_W_18_Ce_3_.

### 3.2. Characterization of the material

#### 3.2.1. FT-IR spectroscopy

**Fig. 1:**
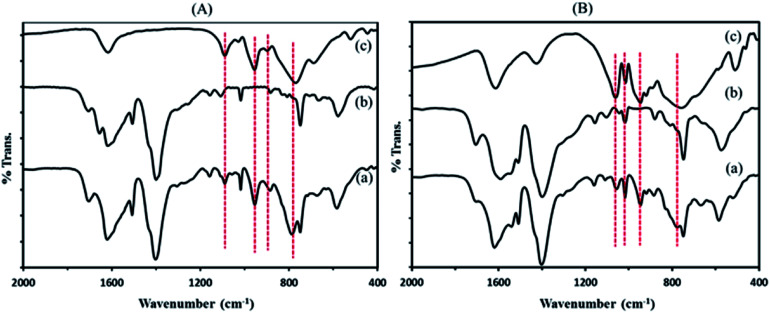
FT-IR spectra of (A) P_2_W_18_Sn_3_@MIL-101 composite (a) the precursors of MIL 101 (b) and P_2_W_18_Sn_3_ (c) and (B) P_2_W_18_Ce_3_@MIL-101 composite (a) the precursors of MIL 101 (b) and P_2_W_18_Ce_3_ (c).

The FT-IR spectra of composite materials and those of the precursor compounds of polyoxometalates and the MIL-101 were compared in Fig. 1. The FT-IR spectra of composites (Fig. 1A(a) and B(a)) exhibit the characteristic bands of both the MIL-101 support (Fig. 1A(b) and B(b)) and sandwich polyoxometalates P_2_W_18_Sn_3_ or P_2_W_18_Ce_3_ (Fig. 1A(c) and B(c)). The new bands at 1093, 956, 891, 792 cm^−1^ or 1062, 1018, 948, 784 cm^−1^ (shown with dashed lines) observed in the spectra of P_2_W_18_Sn_3_@MIL-101 and P_2_W_18_Ce_3_@MIL-101 compared to the that of MIL-101 were attributed to the P–O and W–O vibrations of the sandwich polyoxometalates P_2_W_18_Sn_3_ (ref. 32) or P_2_W_18_Ce_3_,^37^ respectively. The bands in MIL-101, P_2_W_18_Sn_3_@MIL-101 and P_2_W_18_Ce_3_@MIL-101 at 1400 cm^−1^ and 1552 cm^−1^ are correspond to (O–C–O) symmetric vibrations implying the presence of dicarboxylate within the framework, broad and strong bands at around 3430 cm^−1^ and 1620 cm^−1^ also confirms the presence of adsorbed water molecules stretching and bending vibrations or the guest molecules inside the pores and the other weak bands in the spectral region of 600–1600 cm^−1^ are attributed to benzene, including the stretching vibration C=C groups (1514 cm^−1^), in plane and out–of plane bending modes of COO groups (400–700 cm^−1^), and the out–of plane deformation vibrations of benzene ring C–H groups at 1164, 1020, 889, and 748 cm^−1^.^38,39^

#### 3.2.2. Thermogravimetric analysis (TGA)

**Fig. 2:**
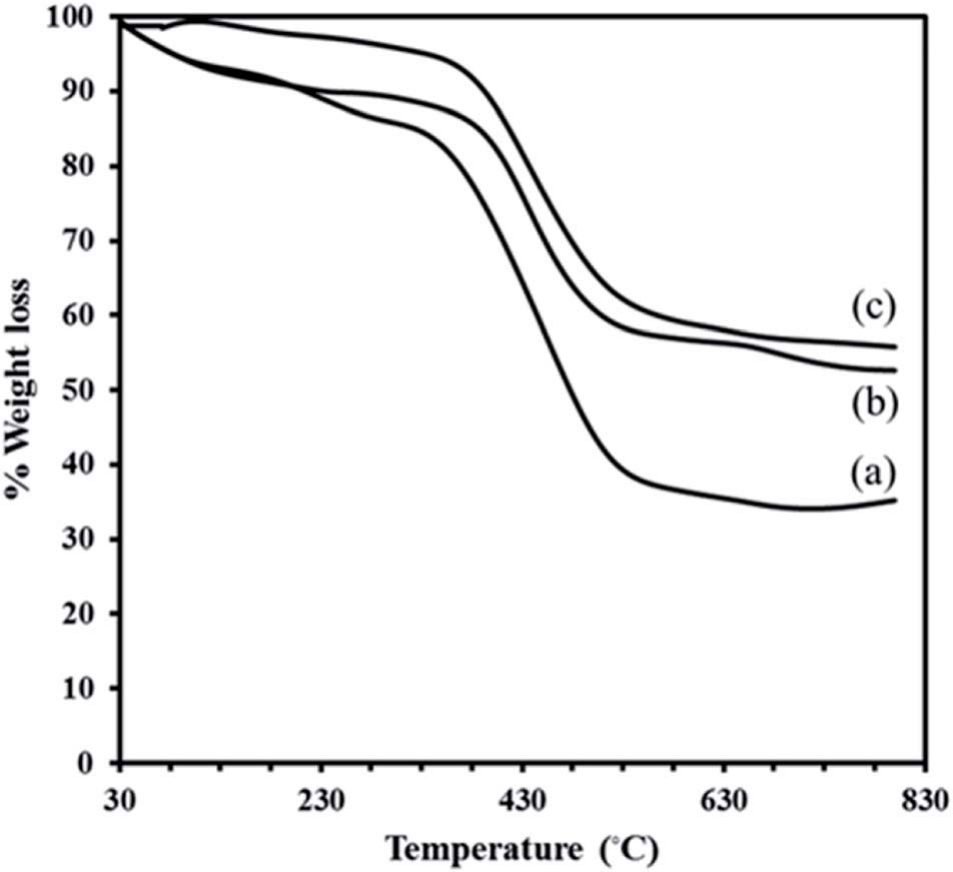
TGA curves of MIL-101 (a), P_2_W_18_Ce_3_@MIL-101 (b) and P_2_W_18_Sn_3_@MIL-101 (c).

The TGA curves for the neat MIL-101 (a) and the composites of P_2_W_18_Ce_3_@MIL-101 (b) and P_2_W_18_Sn_3_@MIL-101 (c) under an inert atmosphere at a constant rate of 10 °C min^−1^ are shown in Fig. 2. A thermal gravimetric analysis study on the pristine MIL-101 showed continuous weight loss in the range of 30–800 °C. Two main weight-loss steps were observed below 400 °C: the first (5.83%), relates to the removal of guest water molecules in the small cages, occurs in the range 30–120 °C; a larger loss of *ca.* 6.51% is due to the departure of chemically bonded water and organic solvent (EtOH/DMF) molecules.^30^ The third step occurs in the range 272–800 °C with loss of about 52.71% relates to the departure of OH/F groups and the decomposition of the framework.

The residual solid is Cr_2_O_3_ for MIL-101. Although, the TGA profile of the composite P_2_W_18_Sn_3_@MIL-101 in the first event, occurs in the range 30–310 °C (2.27%), may be assigned to the removal of water molecules present render in the structure electrostatically neutral or the guest molecules inside the pores. A larger loss of *ca.* 36.91% is due to relates to the departure of OH/F groups and the decomposition of the framework. The minor weight loss of 2.97% in the temperature range 600–800 °C in TGA profile of the composite P_2_W_18_Sn_3_@MIL-101 can attribute to the loss of oxygen atoms from the residual metal oxides resulted from decomposition of P_2_W_18_Sn_3_. The residual mixture solid is Cr_2_O_3_–WO_3_–P_2_O_5_–SnO_2_–K_2_O (55.80%).^40^ Also, the total weight loss of 42.15% for P_2_W_18_Sn_3_@MIL-101 is lower than that for the parent MIL-101 (65.05%), as would be expected from the presence additional residual from partial decomposition of P_2_W_18_Sn_3_ in the cages of composite. The TGA curve of the P_2_W_18_Ce_3_@MIL-101 composite shows a two-stage weight loss below 600 °C, such as the P_2_W_18_Sn_3_@MIL-101 composite, and indicates a lower weight loss than MIL-101. From TGA curves can be recognized that polyoxotungstate anions improve the thermal stability of the neat MIL-101.^41^

#### 3.2.3. SEM and EDX

**Fig. 3:**
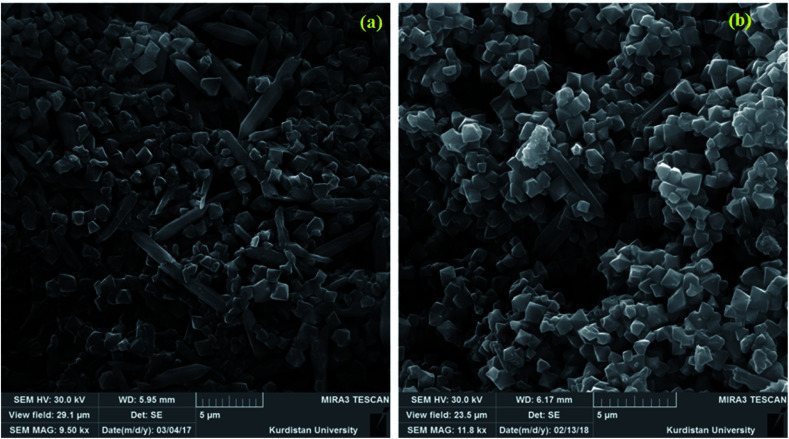
SEM images of MIL-101 (a) and P_2_W_18_Ce_3_@MIL-101 (b).

**Fig. 4:**
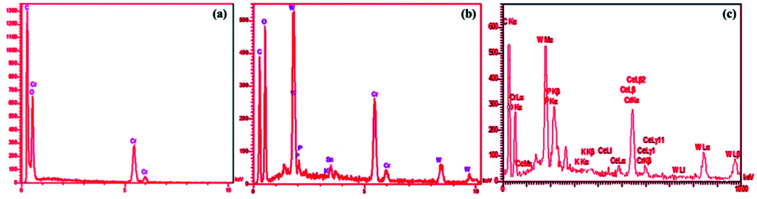
EDX spectra of (a) MIL-101 and the composite of P_2_W_18_Sn_3_@MIL-101 (b) and P_2_W_18_Ce_3_@MIL-101 (c).

The SEM image of the MIL-101 was displayed frequently octahedral with some hexagonal MOF rods (Fig. 3a). As depicted in Fig. 3b, the SEM image the P_2_W_18_Ce_3_@MIL-101 composite material appears like an aggregation state of nano crystallites with the similar morphology of the solid support MIL-101, pointing to the preservation of structure the solid support after the incorporation of P_2_W_18_Ce. EDX analyses of MIL-101 and nanocomposites materials of P_2_W_18_Sn_3_@MIL-101 and P_2_W_18_Ce_3_@MIL-101 demonstrate that all of the elements of MIL-101 and the polyoxometalate anions in the samples which it confirms the presence of the polyoxotungstate anions in POMs@MIL-101 (Fig. 4).

#### 3.2.4. N_2_ adsorption

**Fig. 5:**
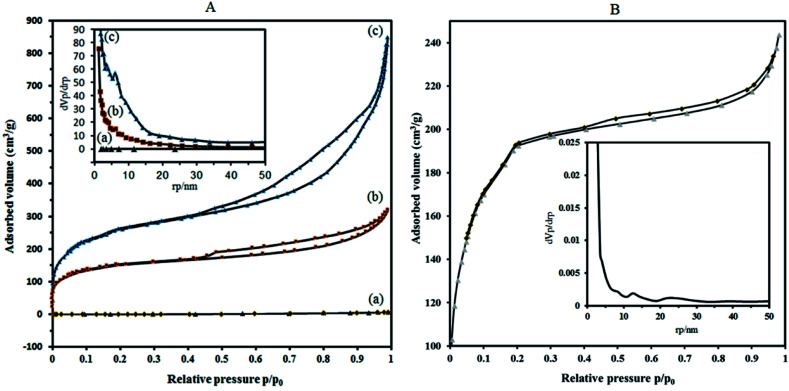
N_2_ adsorption–desorption isotherms at 77 K of (A) P_2_W_18_Sn_3_ (a), the composite P_2_W_18_Sn_3_@MIL 101 (b) and MIL 101 (c). (B) The composite P_2_W_18_Ce_3_@MIL 101. Inset shows BJH pore-size distribution plots of the samples.

Chromium terephthalates contained significant amounts of free guest molecules (solvent or other chemicals used during the synthesis for example; terephthalic acid or DMF) within the pores. To evacuate the free molecules from the MOF with compromising its structural integrity and hence porosity, two effective activation steps were performed. The exchange of the high-boiling point solvent, (*e.g.*, DMF), used for purification, by a lower boiling point solvent (*e.g.*, EtOH) followed by simple heat and vacuum treatment.^42^ DMF molecules were completely removed from the MIL-101 after solvent exchange for one day and activation at 70 °C for 3 days under vacuum. After post-treatment, the pore textural properties enhanced for the as-synthesized MIL-101. Nitrogen adsorption isotherms for P_2_W_18_Sn_3_, MIL-101 (activated with ethanol), P_2_W_18_Sn_3_@MIL-101 and P_2_W_18_Ce_3_@MIL-101 are shown in Fig. 5 at boiling temperature (77 K) after evacuating guest molecules from the samples at 100 °C for overnight. As noted above, the major disadvantages of POMs as catalyst are low surface areas as well as POMs in the bulk phase display no characteristic porosity which limit their utility in many catalytic reactions.

To overcome these problems, it is proposed that increasing the surface area can be achieved by the deposition of sandwich POM into porous solid supports with high surface area. The results of the BET analysis show that the specific surface area of P_2_W_18_Sn_3_ is much lower compared to composite (Fig. 5A(a)).

Nitrogen adsorption isotherms show that the specific surface area of the dehydrated MIL-101 decrease from 946.91 m^2^ g^−1^ to 573.08 m^2^ g^−1^ for P_2_W_18_Sn_3_@MIL-101. Importantly, compared with MIL-101, P_2_W_18_Sn_3_@MIL-101 shows a smaller pore volume (1.312 cm^3^ g^−1^ to 0.491 cm^3^ g^−1^), which may help to interpret the lower specific surface area for composite. Combined with N_2_ sorption isotherms analysis and the pore size distribution (PSD) results, it can be concluded that the doping of the MIL-101 porous structure with POM ions can change morphological characteristics (surface area, pore volume and pore size distributions).^43,44^

Adsorption isotherms of P_2_W_18_Sn_3_@MIL-101 (Fig. 5A(b)), as well as MIL-101 (Fig. 5A(c)), reveal typical type-I behavior with remarkable H_4_ hysteresis loop which is coincident with the mesoporous structures. This hysteresis is usually characteristic of solids consisting of aggregates of particles forming slit-shaped pores (plates or edged particles like cubes), with uniform or non-uniform size and/or shape. These results are in good agreement with the results of the pore size distribution (calculated by BJH method based on the adsorption branch) of P_2_W_18_Sn_3,_ the MIL-101 and P_2_W_18_Sn_3_@MIL-101 materials. From the distribution curves, the samples have a broad pore-size distribution in the range of 1.2–30 nm (Fig. 5 inset).

The N_2_ adsorption isotherm and the BJH pore size distribution curve of P_2_W_18_Ce_3_@MIL-101 catalyst shown in Fig. 5B. Importantly, compared with the MIL-101, P_2_W_18_Ce_3_@MIL-101 catalyst shows the surface area and total pore volume is reduced to 690.14 m^2^ g^−1^ and 0.376 cm^3^ g^−1^, in agreement with the presence of heavy polyoxometalate moieties. According to the results of the Barrett–Joyner–Halenda (BJH) analysis, mesoporous structure are present in both nanocomposites, especially in P_2_W_18_Sn_3_@MIL-101 catalyst, in agreement with its hysteresis loop at relative pressure (*P*/*P*_0_) between 0.4 and 1.0.^45^ The results of the N_2_ adsorption–desorption isotherms and pore size distributions of the synthesized nanocomposites show that the type of polyoxometalate loaded in the cavities of the MIL-101 affects the volume and size distribution of the pores in the material.

#### 3.2.5. Powder X-ray diffraction

**Fig. 6:**
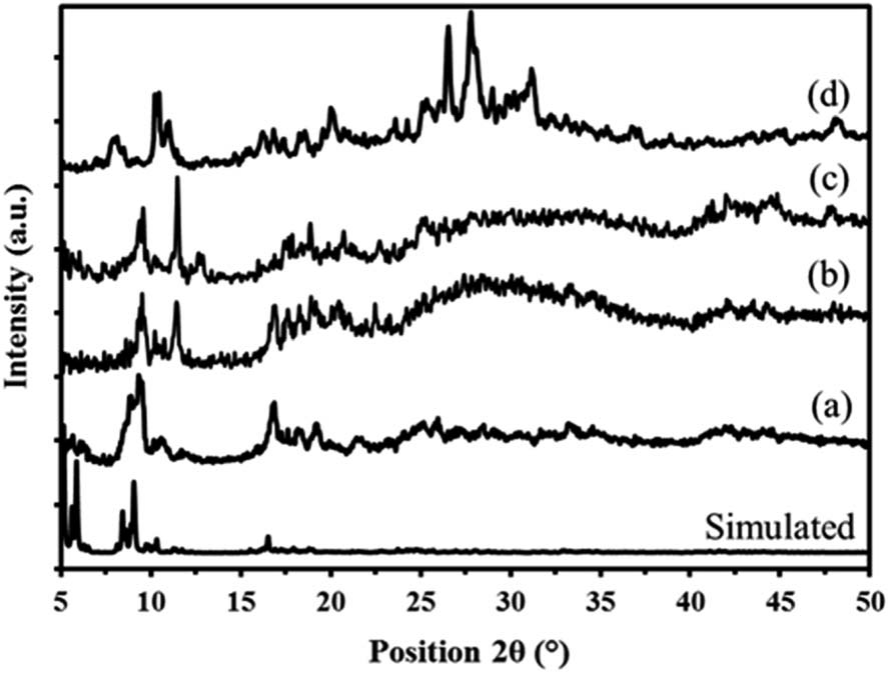
Powder X-ray diffraction patterns of simulated and prepared MIL-101 (a) P_2_W_18_Sn_3_@MIL-101 (b) and P_2_W_18_Ce_3_@MIL-101 (c) sandwich polyoxometalates P_2_W_18_M_3_ (d).

Fig. 6 shows the XRD patterns of MIL-101, P_2_W_18_Sn_3_@MIL-101, P_2_W_18_Ce_3_@MIL-101 and sandwich polyoxometalates P_2_W_18_M_3_ (M = Sn and Ce) in the 2*θ* range of 5–50°. XRD patterns of the obtained MIL-101 metal–organic framework was analyzed referring to the simulated XRD patterns of the MIL-101 single crystal. The simulated XRD patterns of MIL-101 (Fig. 6) exhibited five strong peaks at 2*θ* = 5.2°, 5.6°, 5.9°, 8.4°, 9.1° corresponding to the 511, 440, 351, 822, 911 reflection, respectively.^46,47^ As seen in Fig. 6a, the all of the diffraction peaks corresponding to the obtained MIL-101 are in good agreement with the standard sample. It can see the diffraction peaks of the P_2_W_18_Sn_3_@MIL-101 (Fig. 6b) include both characteristic peaks of MIL-101 and P_2_W_18_Sn_3_ polyoxotungstate (Fig. 6d), revealing the existence of P_2_W_18_Sn_3_ and their basically intact the crystalline characters of the parent metal–organic framework. Powder X-ray diffraction pattern of P_2_W_18_Sn_3_@MIL-101 shows a less-ordered structures pattern which implying that the occupation of pore channels of MIL-101 by polyoxometalate and change in electronic environment around Cr atoms (Fig. 6b).^48^

The X-ray diffraction pattern of the P_2_W_18_Ce_3_@MIL-101 nanocomposite is in good agreement with P_2_W_18_Sn_3_@MIL-101, thus confirming both polyoxotungstates have the same diffraction pattern (Fig. 6c).

### 3.3. Catalytic activity

Organosulfur compounds, such as sulfoxides and sulfones, are useful synthetic intermediates for the construction of various pharmaceutically and biologically active compounds. They are widely utilized as anti-bacterial, anti-fungal, anti-atherosclerotic, anti-ulcer, vasodilators and cardiotonic agents and as well as activation of enzymes. Different synthetic methods for the controlled oxidation of conventional sulfides have been previously reported for fundamental transformation. Traditionally, these transformations take place with stoichiometric amounts of electrophilic reagents, such as peracids, dioxiranes, hypochlorites, periodates and highly toxic oxo metal oxidants (NaIO_4_, MnO_2_, CrO_3_, SeO_2_, PhIO, NH_4_MnO_4_ and so on) but, many of these procedures are accompanied by particular disadvantages such as toxic and corrosive oxidants, long reaction times, inconvenient reaction conditions, environmentally unfavorable catalysts (poor recovery of expensive metal catalysts), low yields and the formation of toxic wastes.^49,50^ Various catalyst systems of organocatalysts, acid catalysts, enzymes, metal catalysts and organic–inorganic hybrid solid materials have been used for this reaction with H_2_O_2_ as an oxygen source. Recently, the use of polyoxometalates was promising not only due to bifunctional redox acid catalysts properties but also due to their unusual properties such as high charges, low cost and low environmental impact.^51–53^

The reaction initially performed in the presence of 5.85 mmol of H_2_O_2_ as an oxidant, employing 1 mmol (0.17 mL) diphenyl sulfide and 50 mg of catalyst in CH_3_CN (2.5 mL) at room temperature. In order to find the optimum reaction conditions, the effect of different reaction parameters such as the amount of catalyst, the stoichiometry of H_2_O_2_ respect to the catalyst and substrate, solvent, reaction time, different dosages of POM into MIL-101 and the catalytic activities of the different catalysts on the selectivity and conversion of the oxidation reaction were studied. No product was obtained in the absence of any catalyst (Table 1, entry 1). At first, the catalytic performance was evaluated for the selective oxidation of sulfides using MIL-101 support and the POMs (P_2_W_18_Sn_3_ and P_2_W_18_Ce_3_) as homogeneous catalysts in different reaction conditions (Table 1, entries 2–6).

**Table 1:** Optimization of the reaction conditions with respect to the effect of the different catalysts^c^ and solvent on the oxidation of diphenyl sulfide

Entry	Catalyst (mg)	Solvent	Time (min)	Sulfoxide (%)^a^	Sulfone (%)^a^
1^b^	Catalyst free	CH_3_CN	1440	Trace	—
2	MIL-101 (50)	CH_3_CN	1440	40	20
3	P_2_W_18_Sn_3_ (50)	CH_3_CN	1440	35	10
4	P_2_W_18_Sn_3_ (50)	H_2_O	1440	Trace	Trace
5	P_2_W_18_Ce_3_ (50)	H_2_O	1440	Trace	Trace
6	P_2_W_18_Ce_3_ (50)	EtOH	180	—	98
7	Catalyst (1) (50)	CH_3_CN	120	—	98
8	Catalyst (2) (50)	CH_3_CN	35	—	98
9	Catalyst (3) (50)	CH_3_CN	210	—	98
10	Catalyst (4) (50)	CH_3_CN	260	—	98
11	Catalyst (5) (50)	CH_3_CN	320	—	98
12	Catalyst (1) (50)	H_2_O	210	—	98
13	Catalyst (1) (50)	EtOH	220	—	98
14	Catalyst (1) (50)	H_2_O/PEG	220	—	98
15	Catalyst (1) (50)	DMF	220	30	30
16	Catalyst (2) (50)	EtOH	60	—	98
17	Catalyst (2) (50)	H_2_O	900	—	92

a Isolated yield.

b Reaction conditions: diphenyl sulfide (1 mmol), 35% H_2_O_2_ (5.85 mmol), solvent (2.5 mL),catalyst (50 mg), r.t.

c [(HOSn^IV^OH)_3_(PW_9_O_34_)_2_]^12−^ @MIL-101 (1), [(HOCe^IV^OH)_3_(PW_9_O_34_)_2_]^12−^ @MIL-101 (2) [(HOSn^IV^OH)_3_(SiW_9_O_34_)_2_]^14−^ @MIL-101 (3), [WCo_3_(H_2_O)_2_(CoW_9_O_34_)_2_]^12−^ @MIL-101 (4) [WZn_3_(H_2_O)_2_(ZnW_9_O_34_)_2_]^12−^ @MIL-101 (5).

The P_2_W_18_Sn_3_ as a homogeneous catalyst in the presence of hydrogen peroxide in H_2_O or CH_3_CN as solvent unsuitable conversion and selectivity was found even in prolonged reaction time (Table 1 entries 3 and 4). The P_2_W_18_Ce_3_ as a homogeneous catalyst in the presence of hydrogen peroxide, although in H_2_O do not show suitable activity but, it catalyzes selectively the oxidation of sulfides to sulfone in about 180 min in EtOH (Table 1 entries 5 and 6). Although the conversion and the selectivity of P_2_W_18_Ce_3_ in EtOH were good, but there are two problems about its separation and recovery. We found that like some of POMs, the structure of P_2_W_18_Ce_3_ in the presence of hydrogen peroxide was converted to a mixture of peroxopolytungstophosphate species.^54,55^ As shown in Table 1, the oxidation of substrate was carried out using the heterogeneous catalysts. Catalytic oxidation of sulfides was carried out in the presence of the MIL-101 composites of various sandwich-type polyoxometalates.

Within this context, [(HOSn^IV^OH)_3_(PW_9_O_34_)_2_]^12−^@MIL-101, [(OCe^IV^O)_3_(PW_9_O_34_)_2_]^12−^@MIL-101, [(HOSn^IV^OH)_3_(SiW_9_O_34_)_2_]^14−^@MIL-101, [WCo_3_(H_2_O)_2_(CoW_9_O_34_)_2_]^12−^@MIL-101 and [WZn_3_(H_2_O)_2_(ZnW_9_O_34_)_2_]^12−^@MIL-101 composites were prepared and their catalytic activities investigated for the oxidation of diphenyl sulfide by using H_2_O_2_ as an oxidant at room temperature in CH_3_CN (Table 1, entries 7–11). The results in Table 1 clearly show that P_2_W_18_Sn_3_@MIL-101 and P_2_W_18_Ce_3_@MIL-101 showed the highest selectivity for oxidizing of diphenyl sulfide in short reaction time with 98% yield of the corresponding sulfone. Increasing catalysts over 50 mg and also H_2_O_2_ more over than 1 mL doesn't have an appreciable effect on the reaction times. The role of different solvents for the oxidation of diphenyl sulfide was examined. As shown in Table 1; in presence P_2_W_18_Sn_3_@MIL-101 when we used acetonitrile as solvent, the desired product (sulfone) can be obtained in a shorter time (Table 1, entry 7). By using H_2_O, EtOH or H_2_O/PEG as solvent similar catalytic conversion and selectivity obtained but at prolonged reaction time (Table 1, entries 12–14). DMF as solvent, the mixture of sulfoxide and sulfone is obtained (Table 1, entry 15). Generally, the solvent type is chosen based on the reaction kinetic and catalyst structure, so choosing the suitable solvents according to the chemical structure, molecular design and its physical and chemical properties is very important in reaction systems. With regard to the porous catalyst in our study, it was not possible to use solvent-free conditions because reactants have to interact with catalytic active species located in the cavities. It is necessary to mention, the reaction in acetonitrile showed a good reactivity (excellent conversion and selectivity), in our this work. On the other hand, in protic solvents such as water, it is possible that Lewis acid sites (Cr^III^) in the structure of the nanocomposite interact with protons of solvent and thus the cavities are blocked, so it is difficult to insertion–extraction for reactants.

The influence of temperature in the diphenyl sulfide oxidation is illustrated with keeping H_2_O_2_ and substrate amount constant. We found similar results at 25 to 50 °C. The reaction was completed selectively in 60 °C at shorter reaction time. Increasing temperature to 85 °C conversion was completed while the selectivity decreased. P_2_W_18_Ce_3_@MIL-101 the best catalytic activity observed in CH_3_CN and EtOH as solvent (Table 1, entries 8 and 16), while in H_2_O the same catalytic activity observed only at very prolonged time (Table 1, entry 17).

The used polyoxometalates have similar structure and the difference among them is the metal ions in the belt of the used sandwich type polyoxometalates. Therefore, the difference in their catalytic activity can be attributed to type of metal ions. The nanocomposites of P_2_W_18_Sn_3_@MIL-101 and P_2_W_18_Ce_3_@MIL-101 show the best catalytic activity which it can be attributed to the Sn^IV^ or Ce^IV^ ions in their structures. A comparison between the latters, show that the best results obtained with P_2_W_18_Ce_3_@MIL-101. Two aligned reasons can be proposed for preference of P_2_W_18_Ce_3_@MIL-101 composite. First, particular ability of cerium in store/release oxygen as an oxygen storage *via* facile reciprocal transformation of Ce^IV^ and Ce^III^ ions under oxidizing and reducing conditions respectively. Due to this feature, activated oxygen species produced from H_2_O_2_, may be stored on the composite, which in turn, may be responsible for oxidation of sulfide.^56–58^ Second, standard electrode potential for reduction of Ce^IV^ to Ce^III^ in the P_2_W_18_Ce_3_@MIL-101 is very more positive than that Sn^IV^ to Sn^II^ in the P_2_W_18_Sn_3_@MIL-101. As an interesting result, in contrast to homogenous condition, the P_2_W_18_Ce_3_ structure was protected in the P_2_W_18_Ce_3_@MIL-101 composite in the presence of hydrogen peroxide.

To investigate the applicability of this procedure, we carried out oxidation of different types of sulfides under the distinctive reaction conditions and the expected products afforded at high yields in CH_3_CN and H_2_O solvents for P_2_W_18_Sn_3_@MIL-101 (Table 2) and for P_2_W_18_Ce_3_@MIL-101 in EtOH (Table 3). As a result from this study, all used sulfides were oxidized to the corresponding sulfone and also noteworthy that P_2_W_18_Ce_3_@MIL-101 composite shows better catalytic activity. In other word, substrates have high selectively to oxidation of sulfur singly even with the presence of functional groups such as aromatic and aliphatic sulfides. For example, 2-methylthio ethanol contains the hydroxyl group was transformed to the corresponding sulfone compound in high conversion and selectivity without dehydrogenation of the hydroxyl group.

**Table 2:** Selective oxidation of various sulfides to sulfones using H_2_O_2_ catalyzed by P_2_W_18_Sn_3_@MIL-101

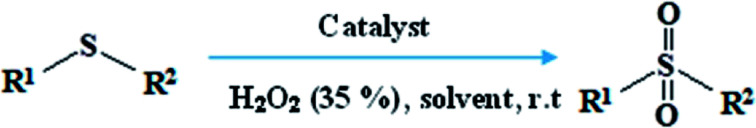					
Entry	Substrate	Sulfone^a^^,^^b^		Sulfone^a^^,^^c^	
		Time (min)	Isolated yield (%)	Time (min)	Isolated yield (%)
1	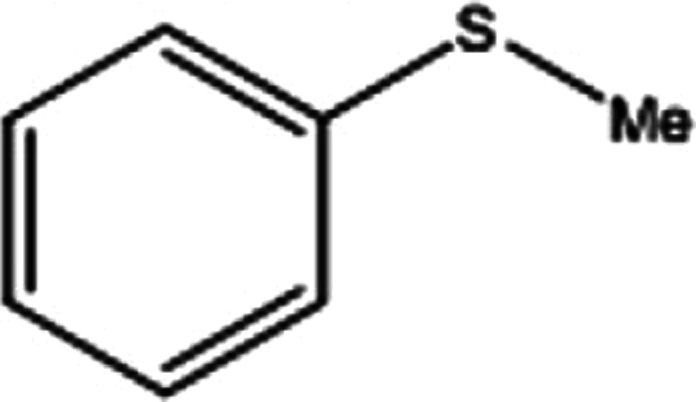	150	98	80	98
2	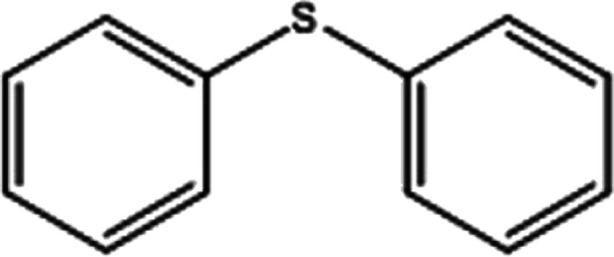	210	98	120	98
3	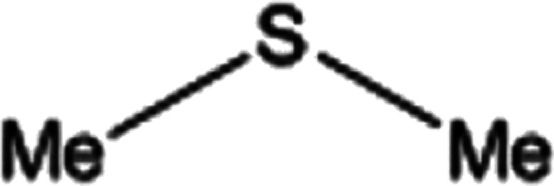	10	97	5	98
4	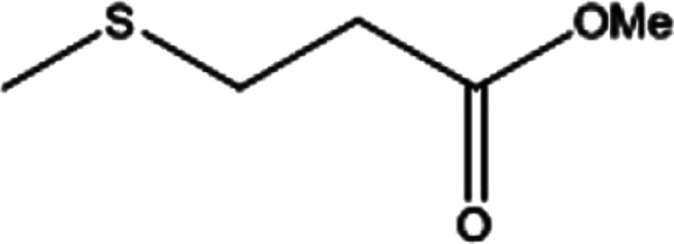	15	95	5	96
5	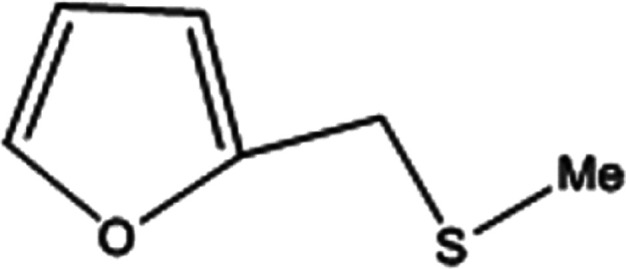	15	97	5	97
6	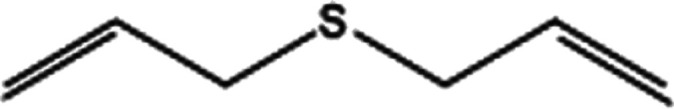	200	94	20	98
7	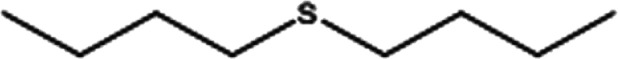	240	94	30	95
8	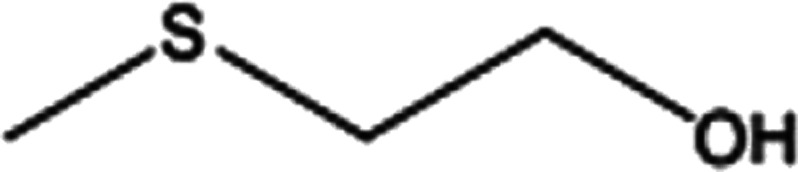	15	96	5	97

a All the products are known and were characterized by FT-IR and by melting point comparison with those of authentic samples.

b Reaction conditions: sulfide (1 mmol), 35% H_2_O_2_ (5.85 mmol), catalyst (50 mg), in H_2_O, r.t.

c Reaction conditions: sulfide (1 mmol), 35% H_2_O_2_ (5.85 mmol), catalyst (50 mg), in CH_3_CN, r.t.

**Table 3:** Selective oxidation of various sulfides to sulfones using H_2_O_2_ catalyzed by P_2_W_18_Ce_3_@MIL-101

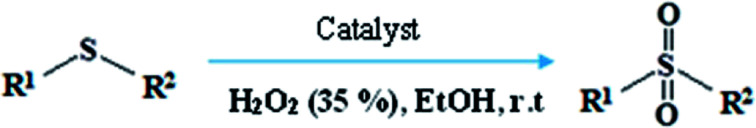			
Entry	Substrate	Sulfone^a^^,^^b^	
		Time (min)	Isolated yield (%)
1	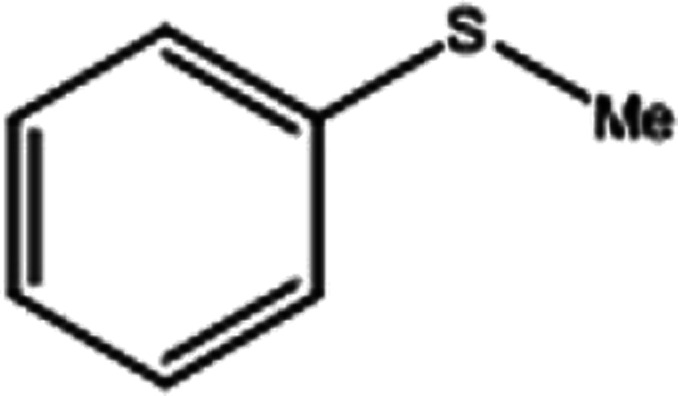	30	98
2	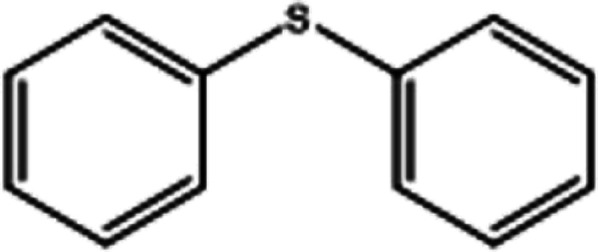	60	98
3	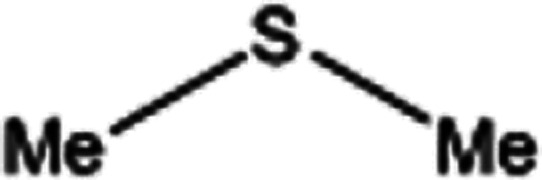	5	97
4	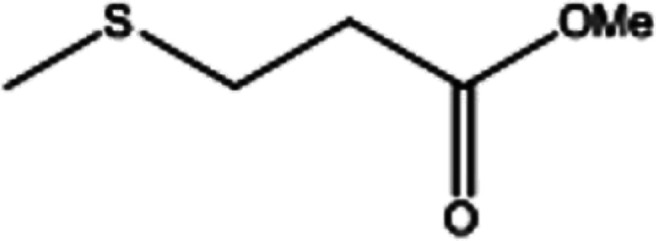	10	96
5	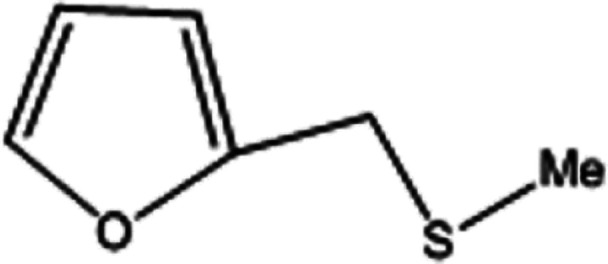	20	97
6	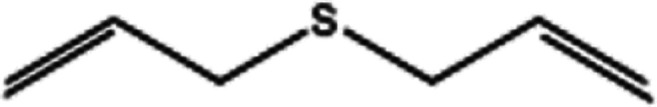	30	96
7	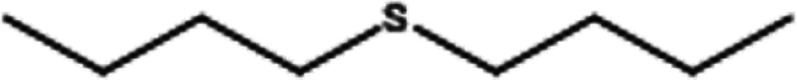	70	94
8	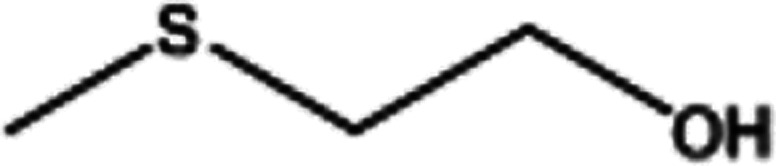	15	96

a All the products are known and were characterized by FT-IR and by melting point comparison with those of authentic samples.

b Reaction conditions: sulfide (1 mmol), 35% H_2_O_2_ (5.85 mmol), catalyst (50 mg), in EtOH, r.t.

### 3.4. Stability of the catalysts

In our final experimental work, stability tests (recovery and reusability) of the new P_2_W_18_Sn_3_@MIL-101 and P_2_W_18_Ce_3_@MIL-101 catalysts were carried out running six consecutive experiments in the oxidation of diphenyl sulfide at a constant time.

**Fig. 7:**
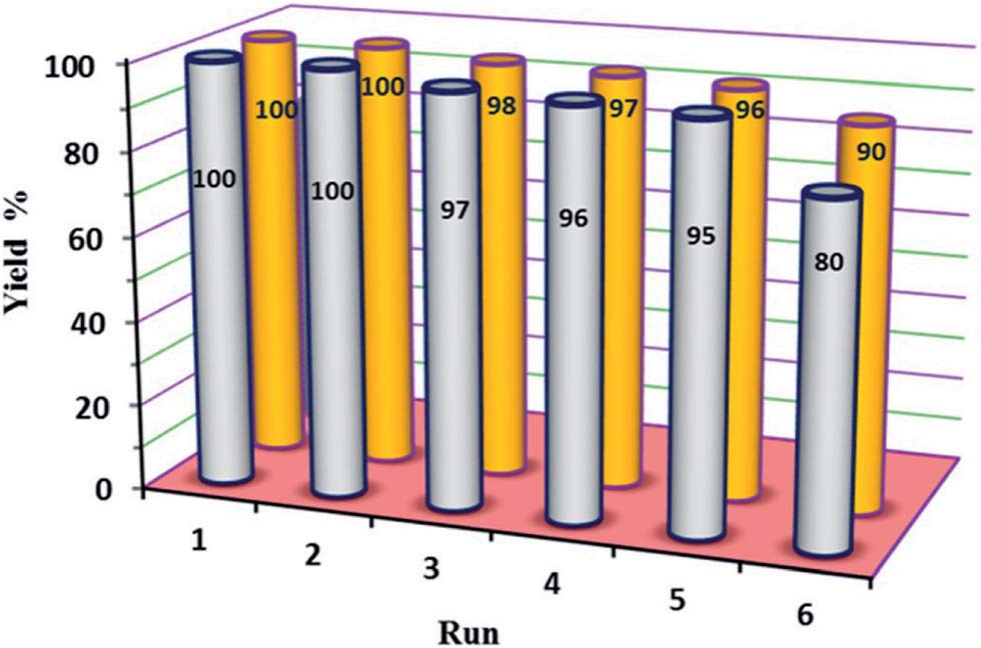
The reusability of P_2_W_18_Sn_3_@MIL-101 (gray) and P_2_W_18_Ce_3_@MIL-101 (orange) catalysts in the oxidation of sulfides.

At the end of each reaction, the catalyst was recovered by simple centrifuge and was washed with ethyl acetate and ethanol (4 × 3 mL), dried under vacuum then used again as the catalyst. The results show the reactions were completed in run of 1–5 at average time of 210 min (in H_2_O) and 60 min (in EtOH) for P_2_W_18_Sn_3_@MIL-101 and P_2_W_18_Ce_3_@MIL-101, respectively. At the same reaction times the 6^th^ runs were completed up to 80 and 90% with P_2_W_18_Sn_3_@MIL-101 and P_2_W_18_Ce_3_@MIL-101 respectively (Fig. 7).

**Fig. 8:**
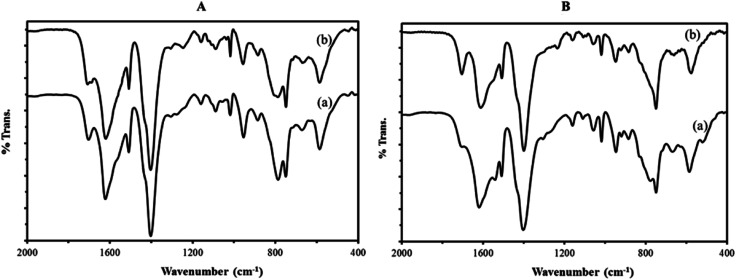
The FT-IR spectra of (A) P_2_W_18_Sn_3_@MIL-101 composite (a) and P_2_W_18_Sn_3_@MIL-101 after six consecutive runs (b) and (B) P_2_W_18_Ce_3_@MIL-101 composite (a) and P_2_W_18_Ce_3_@MIL-101 after six consecutive runs (b).

To investigate the structural stability of catalyst after oxidative reactions, IR spectra fresh and reused nanocomposites were recorded after six catalytic cycles (Fig. 8). The IR spectra of fresh catalyst are identical to those of reused catalyst, confirming the integrity of the support material and also the presence of the sandwich polyoxotungstate anions in the composite material after the catalytic performance.

## 4. Conclusions

Organic–inorganic frameworks based on different sandwich-type polyoxometalates using commercially and readily available materials were synthesized and their catalytic activities studied in the oxidation of different sulfides with an environmentally benign oxidant in different solvents. The nanocomposites have been achieved by impregnation of the synthesized MIL-101 in aqueous solution of the sandwich-type polyoxometalates. This is important point that by increasing concentration of polyoxometalates in aqueous solution, their loading into cages of MIL-101 is not significantly altered. Even after these relatively large anions signing into the MIL-101, there is still enough space to enter the reactants and get out of the product. The POMs insertion into porous MIL-101 solid was investigated using several methods: XRPD, FT-IR, SEM-EDX, ICP, TGA and N_2_ adsorption. Catalysts have a useful life and as a result, with time, their activities and their effect on the reaction are reduced. This means that there are active points on the catalyst, which change over time due to different reasons. So today, the ability to recover and recycle catalyst from the environment is very important and significant. In synthesized composites, these active sites include polyoxometalates that are stable in the reaction media and do not suffer from it. The proposed mechanism for sulfides oxidation over polyoxometalates involves formation of peroxo-tungstate species (an electrophilic intermediate) by the interaction of hydrogen peroxide with the polyoxometalate anions which this transformation can oxidize the sulfides into sulfones. Therefore, in composites containing polyoxometalates, these are mainly catalytically active species that act as a catalyst. The best results were obtained by organic–inorganic framework incorporating of [(HOSn^IV^OH)_3_(PW_9_O_34_)_2_]^12−^ and [(OCe^IV^O)_3_(PW_9_O_34_)_2_]^12−^ among different sandwich polyoxometalates. The results of the BET and TGA analyses show that P_2_W_18_Sn_3_@MIL-101 and P_2_W_18_Ce_3_@MIL-101 catalysts have lower specific surface area than MIL-101, while they show significant higher thermal stability. The advantages of this catalytic synthesis method it can be pointed out to low cost and availability of raw materials, easy synthesis and chemical and thermal stability.

## Conflicts of interest

The authors declare that they have no competing interests.

## Supplementary Material

## References

[cit1] Zhao S., Chen Y., Song Y.-F. (2014). Appl. Catal., A.

[cit2] Mijares K. , Novel hybrid materials: Functionalized polyoxometalates as potential metalloligands, Kansas State University, Germany, 2008

[cit3] Proust A., Matt B., Villanneau R., Guillemot G., Gouzerh P., Izzet G. (2012). Chem. Soc. Rev..

[cit4] Marchenaa C. L., Gomeza S., Sauxa C., Pierellaa L. B., Pizziob L. R. (2015). Quim. Nova.

[cit5] Ghanbaripour R., Mohammadpoor-Baltork I., Moghadam M., Khosropour A. R., Tangestaninejad S., Mirkhani V. (2012). Polyhedron.

[cit6] Zheng X., Zhang L., Li J., Luo S., Cheng J.-P. (2011). Chem. Commun..

[cit7] Guo Y., Hu C., Jiang C., Yang Y., Jiang S., Li X., Wang E. (2003). J. Catal..

[cit8] Villanneau R., Marzouk A., Wang Y., Djamaa A. B., Laugel G., Proust A., Launay F. (2013). Inorg. Chem..

[cit9] Duan X., Liu Y., Zhao Q., Wang X., Li S. (2013). RSC Adv..

[cit10] Kooti M., Afshari M. (2012). Mater. Res. Bull..

[cit11] Afshari M., Gorjizadeh M., Afshar G. (2014). Orient. J. Chem..

[cit12] Tan R., Liu C., Feng N., Xiao J., Zheng W., Zheng A., Yin D. (2012). Microporous Mesoporous Mater..

[cit13] Rafiee E., Eavani S. (2011). Green Chem..

[cit14] Rahimizadeh M., Rajabzadeh G., Khatami S.-M., Eshghi H., Shiri A. (2010). J. Mol. Catal. A: Chem..

[cit15] Han J., Wang D., Du Y., Xi S., Chen Z., Yin S., Zhou T., Xu R. (2016). Appl. Catal., A.

[cit16] Kaskel S. , The chemistry of metal–organic frameworks: synthesis, characterization, and applications, John Wiley & Sons, Germany, 2016

[cit17] Butova V. V. e., Soldatov M. A., Guda A. A., Lomachenko K. A., Lamberti C. (2016). Russ. Chem. Rev..

[cit18] Wu C.-D., Hu A., Zhang L., Lin W. (2005). J. Am. Chem. Soc..

[cit19] Lim S., Suh K., Kim Y., Yoon M., Park H., Dybtsev D. N., Kim K. (2012). Chem. Commun..

[cit20] Abednatanzi S., Abbasi A., Masteri-Farahani M. (2015). J. Mol. Catal. A: Chem..

[cit21] Taylor-Pashow K. M., Rocca J. D., Xie Z., Tran S., Lin W. (2009). J. Am. Chem. Soc..

[cit22] Huang Y.-F., Liu M., Wang Y.-Q., Li Y., Zhang J.-M., Huo S.-H. (2016). RSC Adv..

[cit23] Hong D. Y., Hwang Y. K., Serre C., Ferey G., Chang J. S. (2009). Adv. Funct. Mater..

[cit24] Jhung S. H., Lee J. H., Yoon J. W., Serre C., Férey G., Chang J. S. (2007). Adv. Mater..

[cit25] Biswas S., Couck S., Grzywa M., Denayer J. F., Volkmer D., Van Der Voort P. (2012). Eur. J. Inorg. Chem..

[cit26] Férey G., Mellot-Draznieks C., Serre C., Millange F., Dutour J., Surblé S., Margiolaki I. (2005). Science.

[cit27] Bromberg L., Hatton T. A. (2011). ACS Appl. Mater. Interfaces.

[cit28] Bromberg L., Diao Y., Wu H., Speakman S. A., Hatton T. A. (2012). Chem. Mater..

[cit29] Babahydari A. K., Fareghi-Alamdari R., Hafshejani S. M., Rudbari H. A., Farsani M. R. (2016). J. Iran. Chem. Soc..

[cit30] Julião D., Gomes A. C., Pillinger M., Cunha-Silva L., de Castro B., Gonçalves I. S., Balula S. S. (2015). Fuel Process. Technol..

[cit31] Haddadi H., Hafshejani S. M., Farsani M. R. (2015). Catal. Lett..

[cit32] Bahrami L., Khoshnavazi R., Rostami A. (2015). J. Coord. Chem..

[cit33] Xin F., Pope M. T. (1996). J. Am. Chem. Soc..

[cit34] Knoth W., Domaille P., Harlow R. (1986). Inorg. Chem..

[cit35] Ribeiro S., Granadeiro C. M., Silva P., Paz F. A. A., de Biani F. F., Cunha-Silva L., Balula S. S. (2013). Catal. Sci. Technol..

[cit36] Salomon W., Yazigi F.-J., Roch-Marchal C., Mialane P., Horcajada P., Serre C., Haouas M., Taulelle F., Dolbecq A. (2014). Dalton Trans..

[cit37] Ghiasi Moaser A., Khoshnavazi R. (2017). New J. Chem..

[cit38] Farzaneh F., Sadeghi Y. (2015). J. Mol. Catal. A: Chem..

[cit39] Liu Q., Ning L., Zheng S., Tao M., Shi Y., He Y. (2013). Sci. Rep..

[cit40] Briand L. E., Thomas H. J., Baronetti G. T. (2000). Appl. Catal., A.

[cit41] Abednatanzi S., Leus K., Derakhshandeh P. G., Nahra F., De Keukeleere K., Van Hecke K., Van Driessche I., Abbasi A., Nolan S. P., Der Voort P. V. (2017). Catal. Sci. Technol..

[cit42] Mondloch J. E., Karagiaridi O., Farha O. K., Hupp J.
T. (2013). CrystEngComm.

[cit43] Hu X., Lu Y., Dai F., Liu C., Liu Y. (2013). Microporous Mesoporous Mater..

[cit44] Leofanti G., Padovan M., Tozzola G., Venturelli B. (1998). Catal. Today.

[cit45] Martínez N. D., Venturini R. B., Silva H. S., González J. E., Rodríguez A. M. (2009). Mater. Res..

[cit46] Zhang Y., Wan J., Wang Y., Ma Y. (2015). RSC Adv..

[cit47] Lebedev O., Millange F., Serre C., Van Tendeloo G., Férey G. (2005). Chem. Mater..

[cit48] Islam D. A., Chakraborty A., Acharya H. (2016). New J. Chem..

[cit49] Saux C., Marchena C. L., Pizzio L. R., Pierella L. B. (2016). J. Porous Mater..

[cit50] Chen T.-H., Kwong K. W., Lee N. F., Ranburger D., Zhang R. (2016). Inorg. Chim. Acta.

[cit51] Haddadi H., Farsani M. R. (2016). J. Cluster Sci..

[cit52] Dabiri M., Koohshari M., Shafipour F., Kasmaei M., Salari P., MaGee D. (2016). J. Iran. Chem. Soc..

[cit53] Santos I. C., Gamelas J. A., Duarte T. A., Simões M. M., Neves M. G. P., Cavaleiro J. A., Cavaleiro A. M. (2017). J. Mol. Catal. A: Chem..

[cit54] Zhao W., Yang C. (2013). New J. Chem..

[cit55] Xue X., Zhao W., Ma B., Ding Y. (2012). Catal. Commun..

[cit56] Veisi H., Eshbala F. H., Hemmati S., Baghayeri M. (2015). RSC Adv..

[cit57] Jin Y., Li N., Liu H., Hua X., Zhang Q., Chen M., Teng F. (2014). Dalton Trans..

[cit58] Zou L., Wang Q., Wang Z., Jin L., Liu R., Shen X. (2013). Ind. Eng. Chem. Res..

